# Supplementary material for: Inhibition of defect-induced α-to-δ phase transition for efficient and stable formamidinium perovskite solar cells
Source: Nat Commun. 2023 Sep 30;14:6125. doi: 10.1038/s41467-023-41853-y (PMC10543379; doi:10.1038/s41467-023-41853-y)
Supplement: Supplementary file 1 — Supplementary Information [file 41467_2023_41853_MOESM1_ESM.pdf]

Supplementary Information

Inhibition of defect-induced α-to-δ phase transition for efficient and stable formamidinium perovskite solar cells

Tian Chen[1,2], Jiangsheng Xie[1,2]*, Bin Wen[1,2], Qixin Yin[1,2], Ruohao Lin[1,2], Shengcai Zhu[1]*, Pingqi Gao[1,2]*

[1]School of Materials, Shenzhen Campus of Sun Yat-sen University, No. 66, Gongchang Road, Shenzhen, Guangdong 518107, P.R. China

[2]Institute for Solar Energy Systems, State Key Laboratory of Optoelectronic Materials and Technologies, Sun Yat-sen University, Guangzhou, 510275, P.R. China

*Corresponding author. xiejsh8@mail.sysu.edu.cn (J. X.); zhushc@mail.sysu.edu.cn (S. Z.); gaopq3@mail.sysu.edu.cn (P. G.).

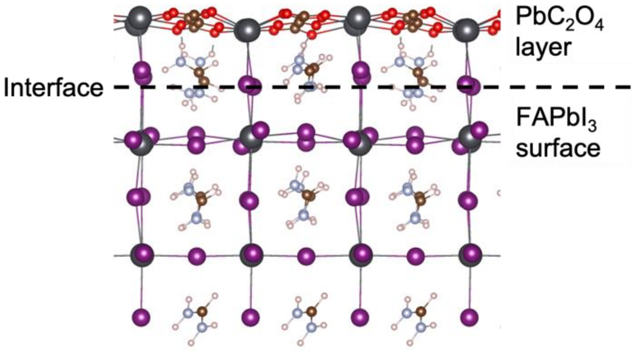

Supplementary Figure 1. The simulation for the interface binding. The binding picture of PbC_2O_4 on $FAPbI_3$ in DFT.

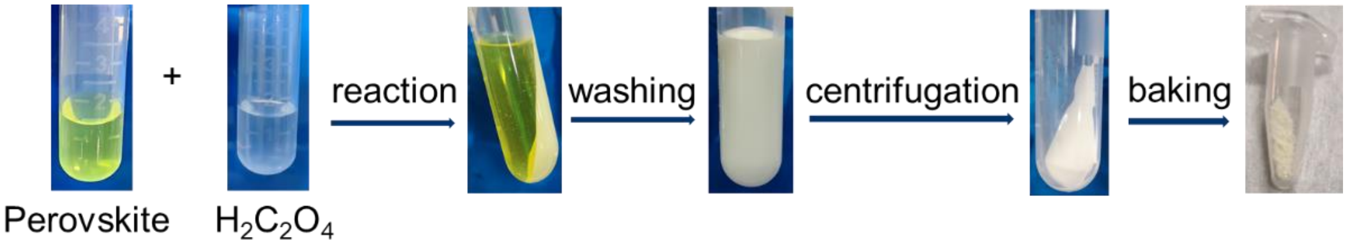

Supplementary Figure 2. The reaction between the oxalic acid and perovskite. Synthetic process of PbC_2O_4 powder by perovskite precursor solution and $H_2C_2O_4$.

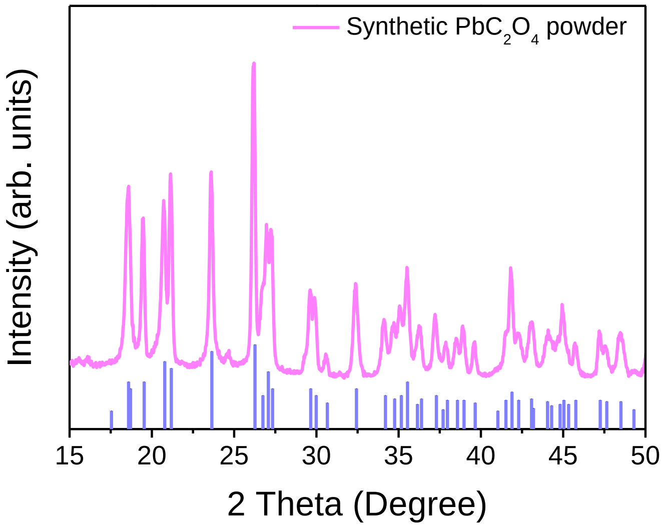

Supplementary Figure 3. Characterization of the synthetic PbC_2O_4 crystallization. XRD spectra of prepared PbC_2O_4 (Synthetic PbC_2O_4 powder, light magenta line). JCPDS: 14-0803 of PbC_2O_4 (light purple line).

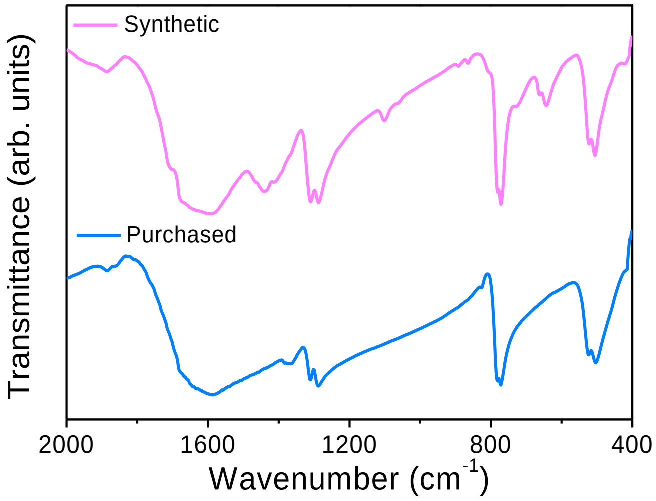

Supplementary Figure 4. The ingredient proof of the synthetic PbC_2O_4. FTIR spectra of the purchased PbC_2O_4 powder (Purchased) and the prepared PbC_2O_4 powder (Synthetic).

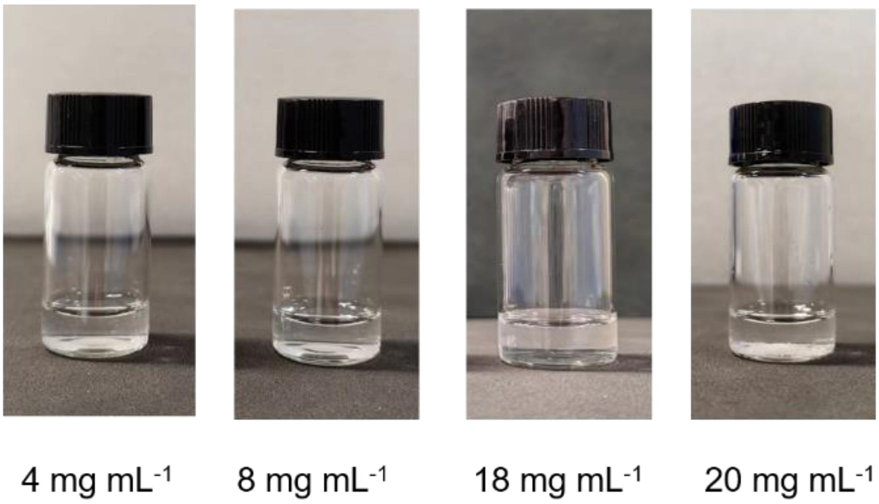

Supplementary Figure 5. The dissolving capacity of $H_2C_2O_4$ in isopropanol. It dissolves well until 18 mg mL^{-1} and some undissolved powder appears at 20 mg mL^{-1}.

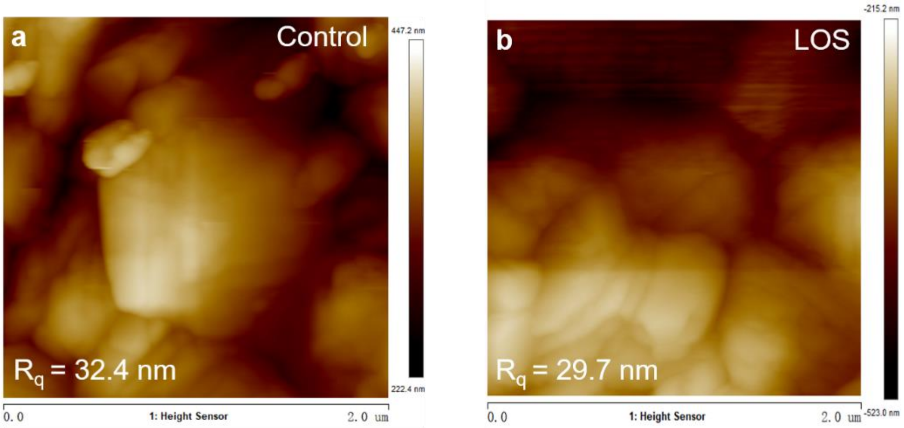

Supplementary Figure 6. The comparison of roughness. AFM images of the (a) control and (b) LOS perovskite films. R_q is root mean square roughness.

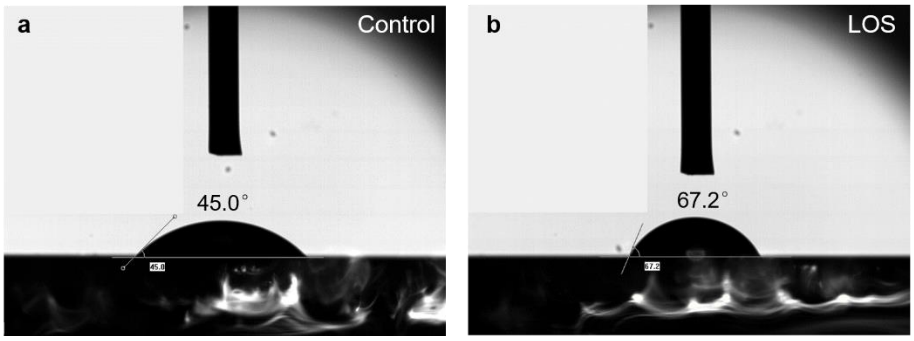

Supplementary Figure 7. The comparison of humidity resistance. Characterization of water contact angles of the (a) control and (b) LOS perovskite films.

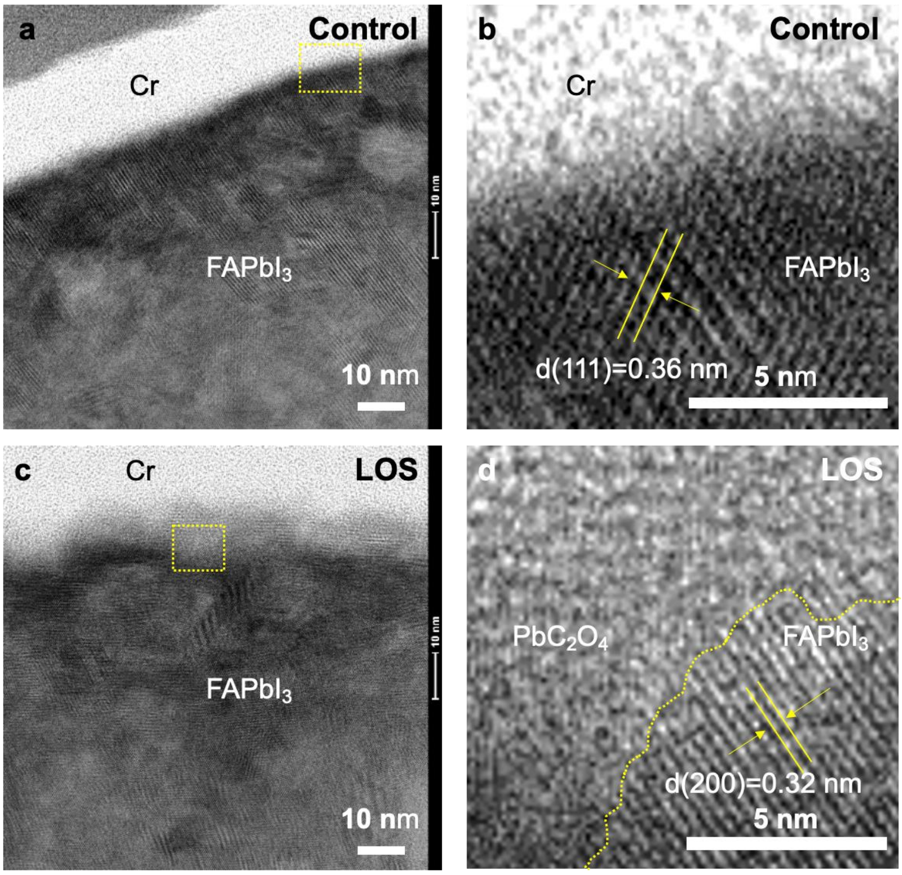

Supplementary Figure 8. The interface between PbC_2O_4 and $FAPbI_3$. HAADF-STEM of (a) control and (c) LOS perovskite. (b) and (d) are the enlargement of the area within the yellow square in (a) and (b), respectively. The inter-planar spacing of 0.36 nm (b) and 0.32 nm (d) matches the (111) and (002) reflection of the cubic α-$FAPbI_3$ perovskite phase, respectively.

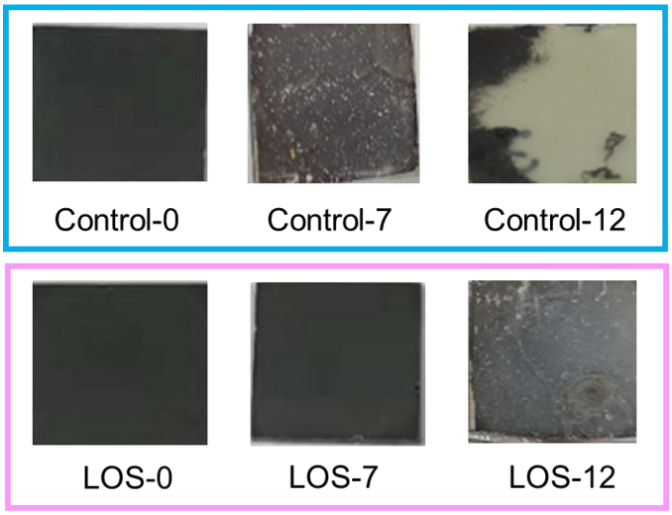

Supplementary Figure 9. Long-term Humidity stability test. The photographs of control and LOS perovskite films stored in 30%-60% relatively humidity after 0 (Control-0 and LOS-0), 7 (Control-7 and LOS-7) and 12 (Control-12 and LOS-12) days.

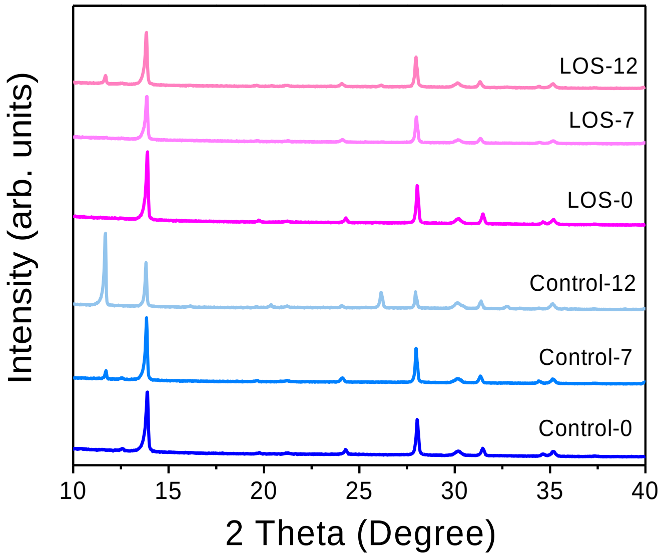

Supplementary Figure 10. Phase transition of the films in long-term humidity stability test. XRD patterns of control and LOS perovskite films stored in 30%-60% relatively humidity after 0 (Control-0 and LOS-0), 7 (Control-7 and LOS-7) and 12 (Control-12 and LOS-12) days.

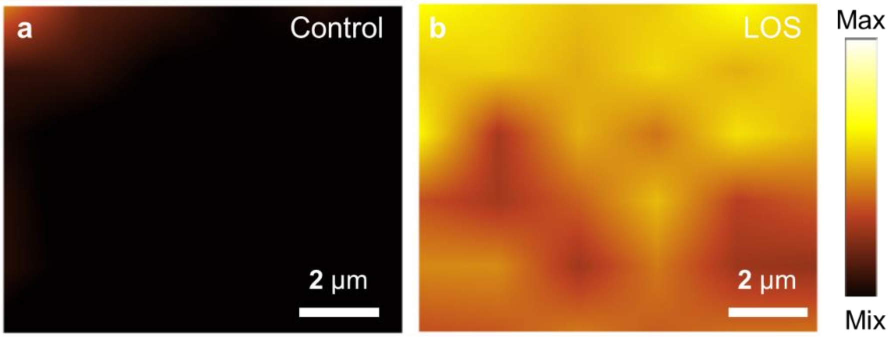

Supplementary Figure 11. PL mapping characteristics. PL mappings of (a) control and (b) LOS films in 10 × 10 μm^2 region.

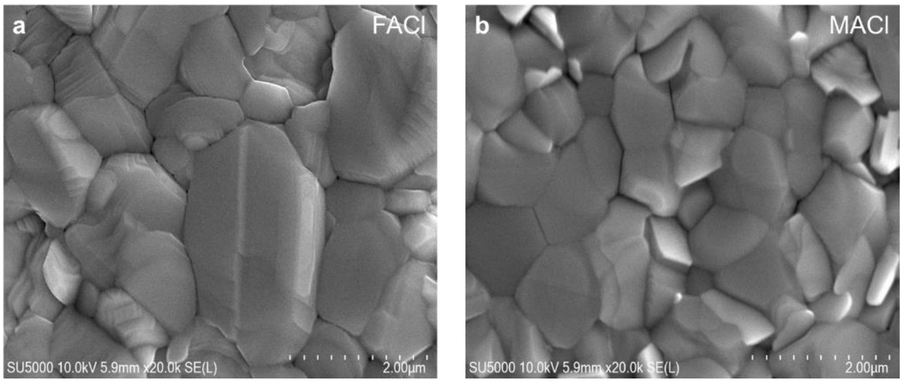

Supplementary Figure 12. **SEM images of the perovskite films**. (a) FACl and (b) MACl as the perovskite precursor additive, respectively. The FACl perovskite film has larger grains than the MACl film.

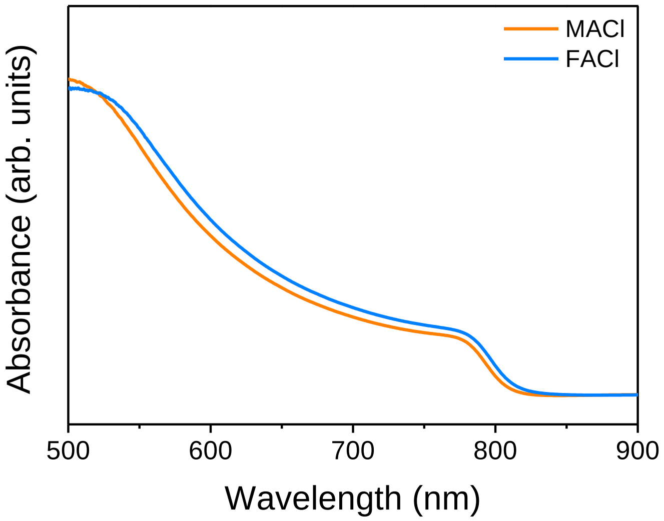

Supplementary Figure 13. UV-vis absorption spectra of the perovskite films with FACl and MACl additive. The FACl perovskite film shows higher absorbance than MACl perovskite and broaden absorption band edge.

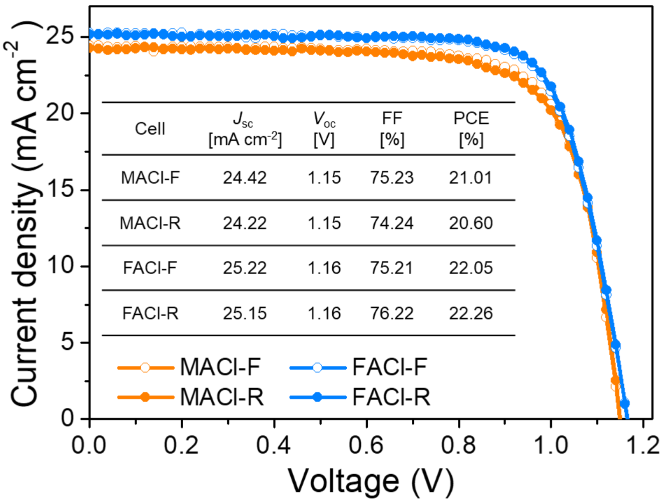

Cell	J_{sc} [mA cm^{-2}]	V_{oc} [V]	FF [%]	PCE [%]
MACl-F	24.42	1.15	75.23	21.01
MACl-R	24.22	1.15	74.24	20.60
FACl-F	25.22	1.16	75.21	22.05
FACl-R	25.15	1.16	76.22	22.26

Supplementary Figure 14. **Device performance with FACl and MACl additive.** Typical *J*-*V* curves of PSCs using FACl and MACl as the perovskite precursor additive, respectively.

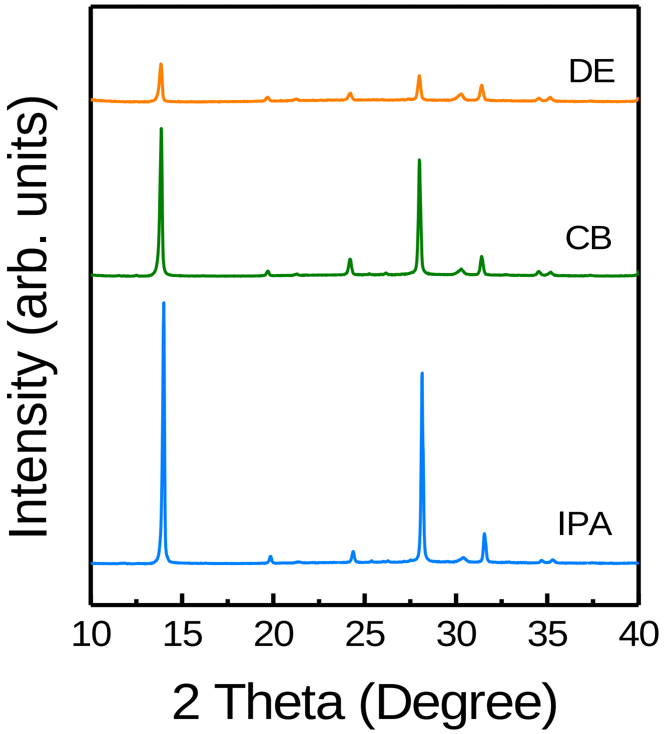

Supplementary Figure 15. The crystallinity of perovskite films with different anti-solvents. XRD of the perovskite films with IPA, CB and DE.

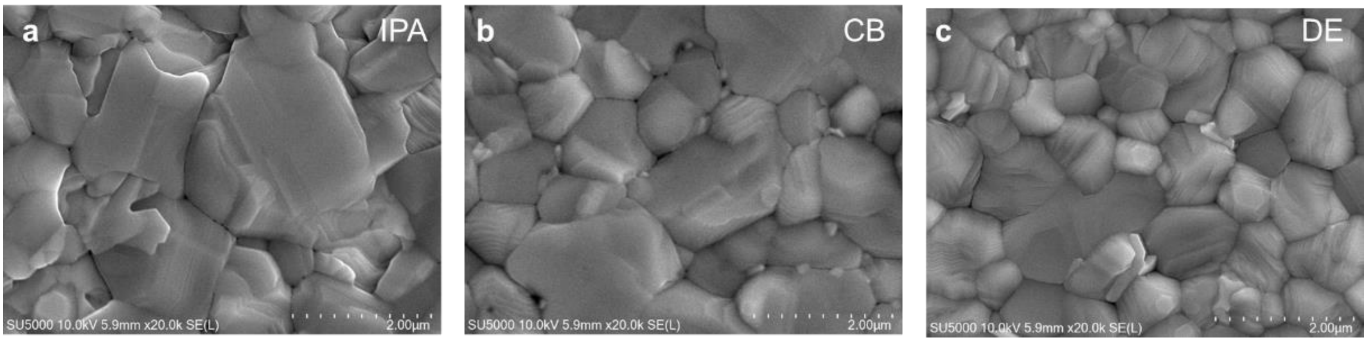

Supplementary Figure 16. The surface morphology characteristics of perovskite films with different anti-solvents. SEM images of the perovskite films with (a) IPA, (b) CB and (c) DE.

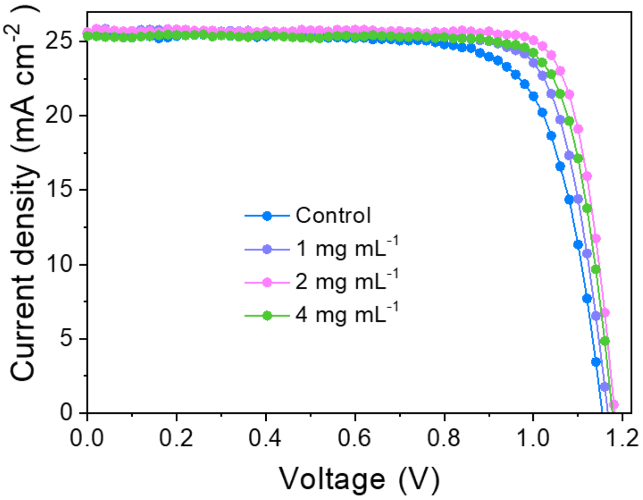

Supplementary Figure 17. Device performance with different concentrations of $H_2C_2O_4$. The *J*-*V* curves of control and LOS PSCs (1, 2, and 4 mg mL^{-1} $H_2C_2O_4$), respectively.

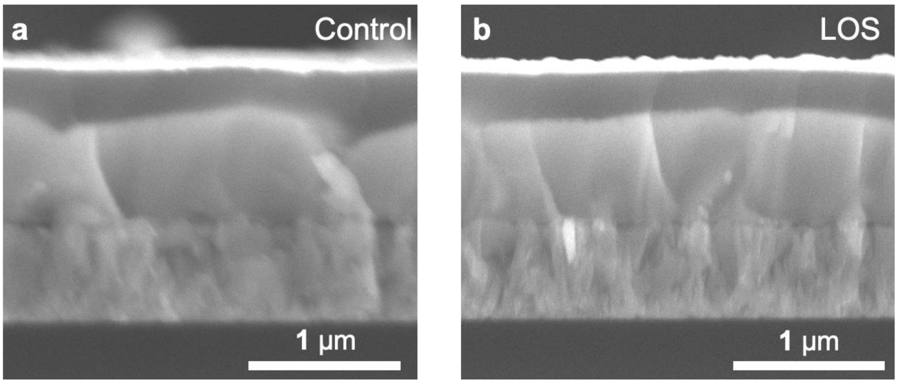

Supplementary Figure 18. **The cross-sectional SEM images**. (a) control and (b) LOS devices.

中国认可
国际互认
检测
TESTING
CNAS L8490
170017163974

Test and Calibration Center of New Energy Device and Module,
Shanghai Institute of Microsystem and Information Technology,
Chinese Academy of Sciences (SIMIT)

Measurement Report

Report No. 23TR022302

**Client Name**	Sun Yat-sen University
**Client Address**	No.66,Gongchang Road,Guangming District,Shenzhen
**Sample**	Perovskite solar cell
**Manufacturer**	Sun Yat-sen University
**Measurement Date**	23th February, 2023

**Performed by:**	Qiang Shi Qiang Shi	**Date:** 23/02/2023
**Reviewed by:**	Wenjie Zhao Wenjie Zhao	**Date:** 23/02/2023
**Approved by:**	Zhengxin Liu Zhengxin Liu	**Date:** 28/2/2023

Address: No.235 Chengbei Road, Jiading, Shanghai
Post Code: 201800
E-mail: solarcell@mail.sim.ac.cn
Tel: +86-021-69976921

The measurement report without signature and seal are not valid.
This report shall not be reproduced, except in full, without the approval of SIMIT.

Report No. 23TR022302

Sample Information

Sample Type	Perovskite Solar Cell
Serial No.	2-2#
Lab Internal No.	23022301-2#
Measurement Item	I-V characteristic
Measurement Environment	24.7±2.0°C, 34.3±5.0%R.H

Measurement of I-V characteristic

**Reference cell**	AK-200(Serial No.:2000041)
**Reference cell Type**	mono-Si, WPVS, calibrated by National Institute of Metrology, China (Certificate No. GXgf2022-01035)
**Calibration Value/Date of Calibration for Reference cell**	128.1mA/ Apr. 2022
**Measurement Conditions**	Standard Test Condition (STC):<br>Spectral Distribution: AM1.5 according to IEC 60904-3 Ed.3,<br>Irradiance: 1000±50W/m^2, Temperature: 25±2°C
**Measurement Equipment/ Date of Calibration**	AAA Steady State Solar Simulator (YSS-T155-2M) / July.2022<br>IV test system (ADCMT 6246) / June. 2022<br>SR Measurement system (CEP-25ML-CAS) / April.2022<br>Measuring Microscope (MF-B2017C) / July.2022
**Measurement Method**	I-V Measurement:<br>Logarithmic sweep in both directions (Voc to Isc and Isc to Voc) during one flash based on IEC 60904-1:2006;<br>Spectral Mismatch factor was calculated according to IEC 60904-7 and I-V correction according to IEC 60891.
**Measurement Uncertainty**	Area: 1.0%(k=2); Isc: 1.8%(k=2); Voc: 1.0%(k=2); Pmax: 2.2%(k=2); Eff: 2.5%(k=2)

Report No. 23TR022302

====Measurement Results ====

	Forward Scan (Isc to Voc)	Reverse Scan (Voc to Isc)
Area	6.76 mm^2	
Isc	1.730 mA	1.732 mA
Voc	1.181 V	1.179 V
Pmax	1.631 mW	1.684 mW
Ipm	1.628 mA	1.656 mA
Vpm	1.002 V	1.017 V
FF	79.81 %	82.52 %
Eff	24.13 %	24.92 %

- Spectral Mismatch Factor SMM=0.9876.
- Designated illumination area defined by a thin blue metal mask was measured by a measuring microscope.
- Test results listed in this measurement report refer exclusively to the mentioned test sample.
- The results apply only at the time of the test, and do not imply future performance.

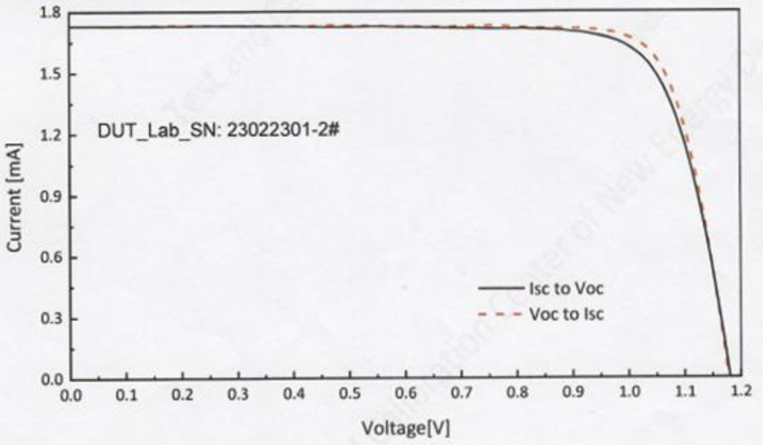

Fig.1 I-V curves of the measured sample

Supplementary Figure 19. Third-party certification. Independent certification by Shanghai Institute of Microsystem and Information Technology (SIMIT) of the LOS device.

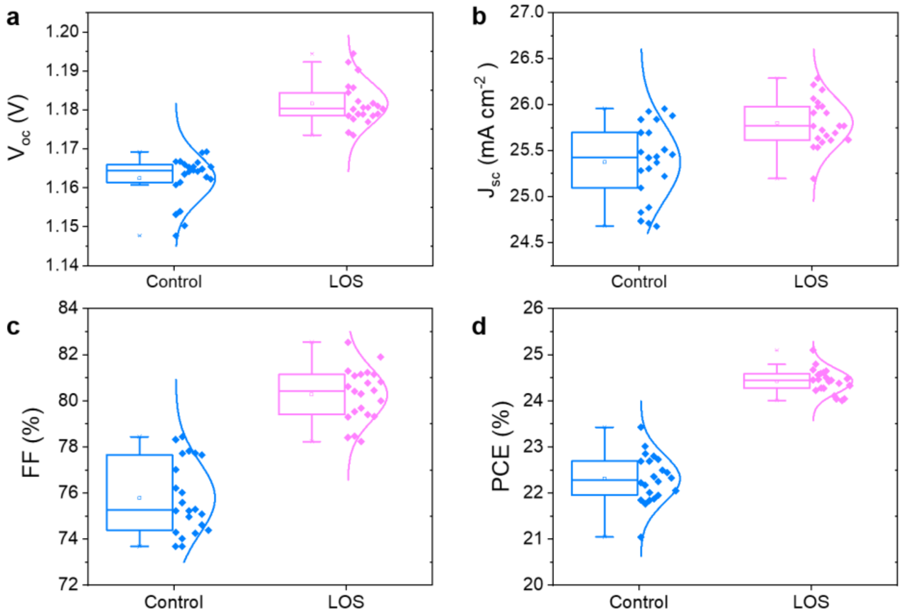

Supplementary Figure 20. The statistical distribution for device performance. (a) V_{oc}, (b) J_{sc}, (c) FF, and (d) PCE of control and LOS perovskite devices ($H_2C_2O_4$, 2 mg mL^{-1}). 22 devices were collected from the different batches. Center line: median, box limits: 25th (the lower) and 75th (the upper) percentile, whiskers: outliers.

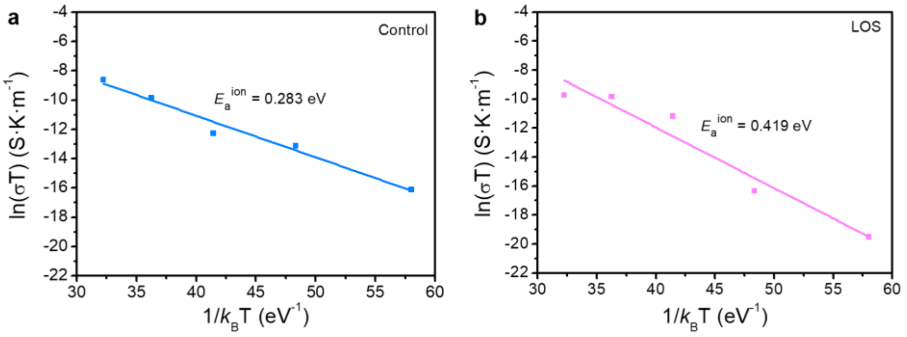

Supplementary Figure 21. The temperature dependent conductivity. The lateral conductivity (σ) changes of (a) control and (b) LOS $FAPbI_3$ films at different temperatures (T) in darkness. E_a^{ion} is activation energy of the ion migration.

Supplementary Table 1. The energy barrier of phase transition and formation energies of native point defects on the surface.

Type	Type Ⅰ			Type Ⅱ			Type Ⅲ		
Defect	V_{Pb}	V_{FA}	I_{Pb}	Pb_I	Pb_{FA}	I_A	V_I	I_i	Pb_i
Energy barrier of phase transition (eV)	0.1	0.36	0.23	0.82	0.89	0.88	0.4	0.46	0.56
Formation energy (eV)	2.32	2.46	2.93	1.87	1.93	2.91	0.69	1.72	1.22

Supplementary Table 2. The formation energies of native point defects on the surface after introducing the PbC_2O_4.

Type	Type Ⅰ			Type Ⅱ			Type Ⅲ		
Defect	V_{Pb}	V_{FA}	I_{Pb}	Pb_I	Pb_{FA}	I_{FA}	V_I	I_i	Pb_i
Formation energy (eV)	2.97	2.76	3.73	1.76	1.73	3.38	1.53	1.92	1.46

Supplementary Table 3. The best photovoltaic parameters of PSCs for the control and LOS devices measured in forward (F) and reverse (R) scan directions under standard AM 1.5 illumination (100 mW cm^{-2}).

Cell	J_{sc} [mA cm^{-2}]	V_{oc} [V]	FF [%]	PCE [%]
Control-F	25.74	1.16	76.69	22.94
Control-R	25.75	1.16	78.40	23.43
LOS-F	25.77	1.18	81.42	24.76
LOS-R	25.77	1.18	83.50	25.39
