# Supplementary material for: Inhibition of defect-induced α-to-δ phase transition for efficient and stable formamidinium perovskite solar cells
Source: Nat Commun. 2023 Sep 30;14:6125. doi: 10.1038/s41467-023-41853-y (PMC10543379; doi:10.1038/s41467-023-41853-y)
Supplement: Supplementary file 2 — Reporting Summary [file 41467_2023_41853_MOESM2_ESM.pdf]

Corresponding author(s): Jiangsheng Xie, NCOMMS-23-25666B

Solar Cells Reporting Summary

Nature Portfolio wishes to improve the reproducibility of the work that we publish. This form is intended for publication with all accepted papers reporting the characterization of photovoltaic devices and provides structure for consistency and transparency in reporting. Some list items might not apply to an individual manuscript, but all fields must be completed for clarity.

For further information on Nature Research policies, including our data availability policy, see Authors & Referees.

Experimental design

Please check the following details are reported in the manuscript, and provide a brief description or explanation where applicable.

1. Dimensions

Area of the tested solar cells

☒ Yes — It can be found in Method section:Device characterization.
☐ No — *Explain why this information is not reported/not relevant.*

Method used to determine the device area

☒ Yes — It can be found in Method section:Device characterization.
☐ No — *Explain why this information is not reported/not relevant.*

2. Current-voltage characterization

Current density-voltage (J-V) plots in both forward and backward direction

☒ Yes — It can be found in Fig. 4c, Supplementary Figs. 14, 19.
☐ No

Voltage scan conditions

☒ Yes — It can be found in Method section:Device characterization.
☐ No — *Explain why this information is not reported/not relevant.*

Test environment

☒ Yes — It can be found in Method section:Device characterization.
☐ No — *Explain why this information is not reported/not relevant.*

Protocol for preconditioning of the device before its characterization

☐ Yes — *Provide a description of the protocol.*
☒ No — No preconditioning protocol was used.

Stability of the J-V characteristic

☒ Yes — We provide continuous maximum power point tracking for 575 hours of unsealed devices in a nitrogen atmosphere under 1-sun illumination without UV filter in Fig. 5a.
☐ No — *Explain why this information is not reported/not relevant.*

3. Hysteresis or any other unusual behaviour

Description of the unusual behaviour observed during the characterization

☒ Yes — In our case, we didn't observed unusual behavior. However, we have provided all forward and reverse scan J-V data for checking hysteresis behavior (Fig. 4c).
☐ No — *Explain why this information is not reported/not relevant.*

Related experimental data

☒ Yes — We have provided hysteresis scans. All other J-V plots were recorded in reverse scan unless not mentioned.
☐ No — *Explain why this information is not reported/not relevant.*

4. Efficiency

External quantum efficiency (EQE) or incident photons to current efficiency (IPCE)

☒ Yes — We have provided EQE spectra in Fig. 4d which was measured by PVE300-IVT210 system (Industrial Vision Technology (s) Pte Ltd). Prior to the use of the light, the spectral response and the light intensity were calibrated using a Si photodiode 300 -1100 nm calibration. All measurements are carried out in ambient environment at ~25 ? and ~35% relative humidity in the dark chamber.
☐ No — *Explain why this information is not reported/not relevant.*

A comparison between the integrated response under the standard reference spectrum and the response measure under the simulator

☒ Yes ☐ No

The integrated short-circuit current from the EQE spectrum is shown in Fig. 4d.

Explain why this information is not reported/not relevant.

For tandem solar cells, the bias illumination and bias voltage used for each subcell

☐ Yes ☒ No

Provide a description of the measurement conditions.

Not applicable.

5. Calibration

Light source and reference cell or sensor used for the characterization

☒ Yes ☐ No

The cells were illuminated using a solar simulator at AM 1.5 G, for which the light intensity was adjusted to 1 sun intensity (100 mW cm-2) through the use of an NREL calibrated Si solar cell.

Explain why this information is not reported/not relevant.

Confirmation that the reference cell was calibrated and certified

☒ Yes ☐ No

The reference cell was firstly calibrated with Si solar cell under standard protocol.

Explain why this information is not reported/not relevant.

Calculation of spectral mismatch between the reference cell and the devices under test

☒ Yes ☐ No

A spectra mismatch calculation was performed based on the spectral irradiation of the solar simulator. The mismatch factor was ~0.9996.

Explain why this information is not reported/not relevant.

6. Mask/aperture

Size of the mask/aperture used during testing

☒ Yes ☐ No

The size of aperture metal mask is 0.0676 cm2, as shown in the certification report (Supplementary Fig. 19).

Explain why this information is not reported/not relevant.

Variation of the measured short-circuit current density with the mask/aperture area

☒ Yes ☐ No

We didn't observed a significant variation.

Explain why this information is not reported/not relevant.

7. Performance certification

Identity of the independent certification laboratory that confirmed the photovoltaic performance

☒ Yes ☐ No

Shanghai Institute of Microsystem and Information Technology (SIMIT)

Explain why this information is not reported/not relevant.

A copy of any certificate(s)

☒ Yes ☐ No

It can be found in Supplementary Fig. 19.

Explain why this information is not reported/not relevant.

8. Statistics

Number of solar cells tested

☒ Yes ☐ No

Statistical data of respective samples is given in Supplementary Fig. 20 for 22 devices in each type.

Explain why this information is not reported/not relevant.

Statistical analysis of the device performance

☒ Yes ☐ No

We have provided statistical data in Supplementary Fig. 20 and Supplementary Data of Source Data.

Explain why this information is not reported/not relevant.

9. Long-term stability analysis

Type of analysis, bias conditions and environmental conditions

☒ Yes ☐ No

The unencapsulated devices were tested at the maximum power point under full-sun illumination in a nitrogen atmosphere to explore its long-term operational stability. We used a electric fan to maintain a relatively constant temperature. More details can be found in "Stability of PSCs".

Explain why this information is not reported/not relevant.
